# Pharmacological cardioversion for atrial fibrillation and flutter*

**Published:** 2006-01-01

**Authors:** J Cordina, G Mead

Atrial fibrillation is the commonest cardiac dysrhythmia.  It is associated with significant morbidity and mortality.  There are two approaches to the management of atrial fibrillation: controlling the ventricular rate or converting to sinus rhythm in the expectation that this would abolish its adverse effects.

The objective of this review was to assess the effects of pharmacological cardioversion of atrial fibrillation in adults on the annual risk of stroke, peripheral embolism, and mortality.

We made a thorough search for existing evidence in the following databases: the Cochrane Controlled Trials Register  (Issue 3, 2002), MEDLINE (2000 to 2002), EMBASE (1998 to 2002), CINAHL  (1982 to 2002), Web of Science (1981 to 2002).  We also handsearched the following journals: Circulation (1997 to 2002), Heart (1997 to 2002),  European Heart Journal (1997-2002), Journal of the American College of Cardiology (1997-2002) and selected abstracts published on the web site of the North American Society of Pacing and Electrophysiology (2001, 2002).  We selected trials based on the following criteria: randomised controlled trials or controlled clinical  trials of pharmacological cardioversion versus rate control in adults (>18  years) with acute, paroxysmal or sustained atrial fibrillation or atrial flutter, of any duration and of any aetiology.

We identified two completed studies AFFIRM (n=4060) and PIAF (n=252). We found no difference in mortality between rhythm control and rate control - relative risk 1.14 (95% confidence interval 1.00 to 1.31).  Both studies show significantly higher rates of hospitalisation and adverse events in the rhythm control group and no difference in quality of life between the two treatment groups. In AFFIRM there was a similar incidence of ischaemic stroke, bleeding and systemic embolism in the two groups. Certain malignant dysrhythmias were significantly more likely to occur in the rhythm control group. There were similar scores of cognitive assessment in both groups. In PIAF, cardioverted patients enjoyed an improved exercise tolerance but there was no overall benefit in terms of symptom control or quality of life.

There is no evidence that pharmacological cardioversion of atrial fibrillation to sinus rhythm is superior to rate control. Rhythm control is associated with more adverse effects and increased hospitalisation. It does not reduce the risk of stroke. These conclusions cannot be generalised to all people with atrial fibrillation as most of the patients included in these studies were relatively older (>60 years) with significant cardiovascular risk factors.

